# Gemcitabine and Platinum-Based Agents for the Prediction of Cancer-Associated Venous Thromboembolism: Results from the Vienna Cancer and Thrombosis Study

**DOI:** 10.3390/cancers12092493

**Published:** 2020-09-03

**Authors:** Florian Moik, Nick van Es, Florian Posch, Marcello Di Nisio, Thorsten Fuereder, Matthias Preusser, Ingrid Pabinger, Cihan Ay

**Affiliations:** 1 Clinical Division of Haematology and Haemostaseology, Department of Medicine I, Comprehensive Cancer Center Vienna, Medical University of Vienna, 1090 Vienna, Austria; florian.moik@meduniwien.ac.at (F.M.); ingrid.pabinger@meduniwien.ac.at (I.P.); 2 Department of Vascular Medicine, Amsterdam Academic Medical Center, 1105 Amsterdam, The Netherlands; n.vanes@amsterdamumc.nl; 3 Division of Oncology, Department of Internal Medicine, Comprehensive Cancer Center Graz, Medical University of Graz, 8036 Graz, Austria; florian.posch@medunigraz.at; 4 Department of Medicine and Ageing Sciences, University G. D’Annunzio, 66100 Chieti, Italy; mdinisio@unich.it; 5 Clinical Division of Oncology, Department of Medicine I, Comprehensive Cancer Center Vienna, Medical University of Vienna, 1090 Vienna, Austria; thorsten.fuereder@meduniwien.ac.at (T.F.); matthias.preusser@meduniwien.ac.at (M.P.); 6 I. M. Sechenov First Moscow State Medical University, 119146 Moscow, Russia

**Keywords:** cancer, venous thromboembolism, chemotherapy, gemcitabine, platinum compounds

## Abstract

**Simple Summary:**

Certain chemotherapy agents (gemcitabine, platinum-based agents) have been suggested to increase the risk of venous thromboembolism in cancer patients. Our aim was to evaluate, whether treatment with these agents can be used to better predict the risk of cancer-associated venous thromboembolism. Within a prospective observational cohort study, including 1409 patients, we found that treatment with gemcitabine and/or platinum-based agents is only of limited value in predicting the risk of venous thromboembolism beyond known risk factors included in an established risk prediction model (tumor type, blood levels of D-dimer). These findings suggest that a large part of the observed rate of venous thromboembolism in patients treated with these agents might be related to the underlying thrombotic risk rather than the agent itself.

**Abstract:**

Gemcitabine and platinum-based agents could increase the risk of venous thromboembolism (VTE) in patients with cancer. We evaluated the additive predictive utility of these agents towards cancer-associated VTE beyond a recently developed and externally validated clinical prediction model, which was based on tumor entity and continuous D-dimer levels. Analysis was performed in the derivation cohort of this model, obtained from the Vienna Cancer and Thrombosis Study (CATS), a prospective observational cohort study (*n* = 1409). Patients were followed for the occurrence of VTE for a maximum of two years. Competing-risk analysis was performed to obtain cumulative incidences and to conduct between-group comparisons of VTE risk. Cumulative two-year incidences of VTE were not elevated with gemcitabine treatment (10.2% vs. 7.5%, *p* = 0.148), whereas they were higher for platinum-based therapy (11.6% vs. 5.9%, *p* < 0.001). In a multivariable analysis, adjusting for tumor site category and D-dimer, gemcitabine was not associated with increased risk of VTE (subdistribution hazard ratio (SHR) 0.82, 95% confidence interval (CI) 0.53–1.28, *p* = 0.390), whereas platinum-based therapy predicted for a numerically increased VTE risk (SHR 1.44, 95% CI 0.96–2.17, *p* = 0.080). Similar results were obtained in a sensitivity analysis (updated cohort, *n* = 1870). Our findings suggest limited additional value of chemotherapy for the prediction of cancer-associated VTE, beyond a validated clinical prediction model.

## 1. Introduction

Various tumor-, patient- and treatment-related factors contribute to the high risk of venous thromboembolism (VTE) in patients with cancer [1]. Among antineoplastic treatments, especially platinum-based agents and gemcitabine have been particularly implicated in increasing risk of cancer-associated thromboembolism [2,3,4]. Consequently, treatment with either platinum-based agents or gemcitabine has previously been incorporated in the PROTECHT-score, a modified version of the Khorana score for the prediction of VTE in patients with cancer [5]. The performance of this score has been evaluated in two independent cohort studies that reported c-indices of 0.59 and 0.61, indicating only moderate discrimination [6,7].

Recently, we developed and externally validated the Vienna Cancer and Thrombosis model (CATS model), a simple clinical prediction model for VTE in patients with cancer based solely on tumor entity and continuous pretherapeutic levels of D-dimer [8]. The aim of the present analysis was to evaluate platinum-based or gemcitabine therapy for the prediction of cancer-associated VTE and to explore the additive predictive value of these variables beyond those incorporated in the validated CATS score.

## 2. Results

### 2.1. Study Cohort

Among the 1409 patients included in the present analysis, 235 (17%) received gemcitabine and 501 (36%) received platinum-based therapy. One-hundred-sixty-seven (12%) patients received both gemcitabine and platinum-based therapy. Importantly, baseline clinical characteristics strongly differed between patients with and without gemcitabine or platinum-based therapy (Table 1). Patients receiving gemcitabine or platinum-based therapy had a significantly higher prevalence of high or very high VTE risk tumor types according to the classification of the CATS score (95% vs. 68% in patients treated with gemcitabine vs. no gemcitabine, respectively, and 99% vs. 58% in patients treated with platinum vs. no platinum, respectively). They also more frequently had higher histological tumor grades (G3/G4 gemcitabine vs. no gemcitabine, 43% vs. 36%, *p* = 0.05 and platinum vs. no platinum, 41% vs. 36%, *p* = 0.04), higher cancer stage (stage IV gemcitabine vs. no gemcitabine, 69% vs. 46%, *p* < 0.001 and platinum vs. no platinum, 67% vs. 40%, *p* < 0.001), and higher levels of D-dimer (median (interquartile range) gemcitabine vs. no gemcitabine, 1.2 (0.6–2.6) vs. 0.7 (0.3–1.3), *p* < 0.001 and platinum vs. no platinum, 1.0 (0.5–2.0) vs. 0.6 (0.3–1.2), *p* < 0.001). Therefore, risk of VTE in patients treated with platinum-based agents or gemcitabine was suspected to be confounded by known strong independent risk factors for cancer-associated VTE [1,9,10].

### 2.2. Crude VTE Incidence According to Chemotherapeutic Agent

**Figure 1:**
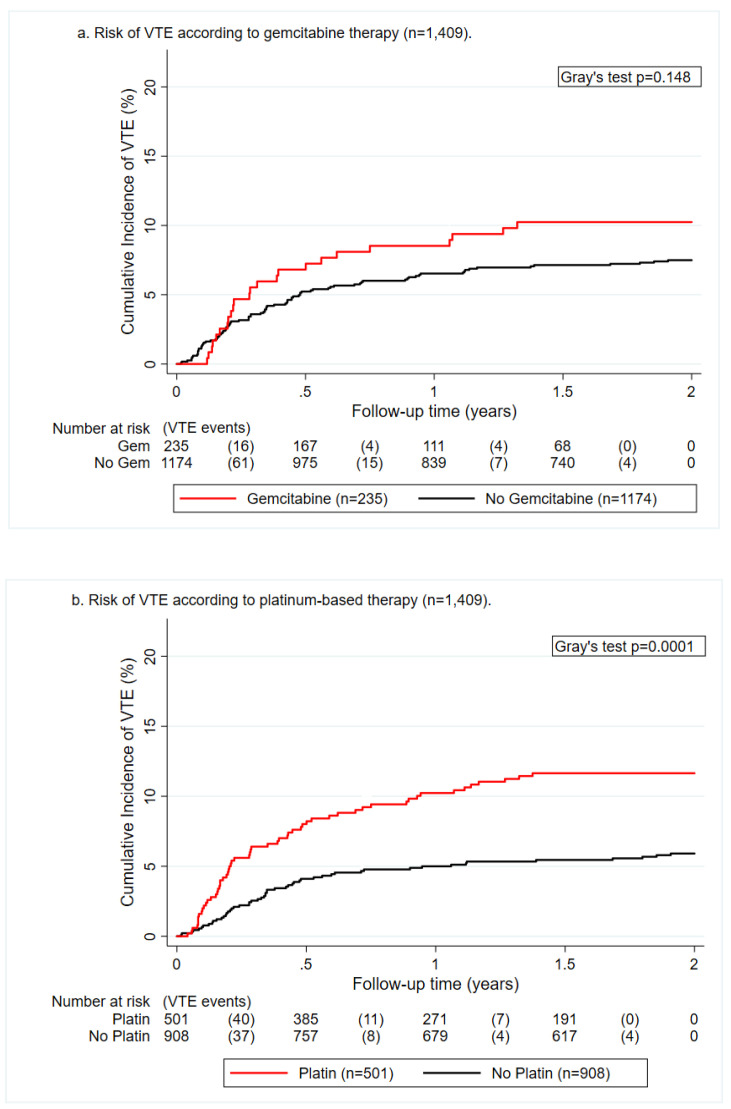
Cumulative incidence function of venous thromboembolism (VTE) risk according to gemcitabine (**a**) and platinum-based chemotherapy (**b**).

Over a median follow-up of 24 months, we observed 111 patients with VTE. Crude two-year cumulative VTE incidences did not significantly differ between patients treated with gemcitabine as compared with those without gemcitabine (10.2%, 95% confidence interval (CI) 6.8–14.5 vs. 7.5%, 95% CI 6.1–9.1, Gray’s test, *p* = 0.148, Figure 1a). The cumulative incidence of VTE at 2 years was significantly higher in patients undergoing platinum-based therapy as compared with those without platinum therapy (11.6%, 95% CI 9.0–14.6 vs. 5.9%, 95% CI 4.5–7.6, *p* < 0.001, Figure 1b). Patients treated with both platinum-based agents and gemcitabine during the study period had a VTE risk that was comparable to the remainder of the patients (9.0%, 95% CI 5.3–14.0 vs. 7.8%, 95% CI 6.4–9.4, *p* = 0.593).

### 2.3. Prediction of Cancer-Associated Thrombosis Beyond the CATS Score

Gemcitabine was not associated with risk of VTE in the univariable analysis (SHR 1.39, 95% CI 0.88–2.18, *p* = 0.156). This association remained similar upon multivariable adjustment for tumor type (SHR 0.83, 95% CI 0.53–1.31, *p* = 0.432), D-dimer (SHR 1.18, 95% CI 0.75–1.85, *p* = 0.481), and tumor type and D-dimer (SHR 0.82, 95% CI 0.53–1.28, *p* = 0.390, Model #1–3 in Table 2). These results suggest a lack of independent predictive utility of gemcitabine therapy towards cancer-associated VTE beyond tumor type or levels of D-dimer.

We observed a strong univariable association between platinum-based therapy and risk of VTE (SHR 2.04, 95% CI 1.41–2.97, *p* < 0.001). Magnitude and strength of association were weakened upon multivariable adjustment for tumor type (SHR 1.46, 95% CI 0.97–2.21, *p* = 0.073), D-dimer (SHR 1.84, 95% CI 1.26–2.69, *p* = 0.002), and both tumor type and d-dimer (SHR 1.44, 95% CI 0.96–2.17, *p* = 0.080, Model #4–6 in Table 2). These findings suggest a potential weak association between platinum-based therapy and risk of cancer-associated VTE beyond tumor type or levels of D-dimer.

### 2.4. Sensitivity Analysis

In order to further explore the putative association of therapy with platinum-based agents or gemcitabine with VTE, a sensitivity analysis was performed with an updated cohort of patients recruited within the Vienna Cancer and Thrombosis study (*n* = 1870). Consistent with the main analysis, therapy with gemcitabine was not associated with risk of VTE in the univariable analysis (SHR 1.17, 95% CI 0.76–1.80, *p* = 0.478). Therapy with platinum-based agents was associated with higher VTE risk in the univariable analysis (SHR 1.50, 95% CI 1.09–2.07, *p* = 0.014). This association was weakened upon adjustment for tumor site category and D-dimer levels in the multivariable analysis (SHR 1.21, 0.86–1.70, *p* = 0.280).

## 3. Discussion

The present findings suggest that platinum-based therapy and gemcitabine do not significantly improve prediction of VTE in patients with cancer beyond an established risk prediction model. Univariable analyses confirmed a relative increase in risk of VTE with platinum-based therapy and gemcitabine as reported previously [2,4]. This concept is further supported by data on vascular toxicity of these agents from preclinical work and clinical observations [11]. Platinum-based agents have been shown to induce endothelial cell dysfunction, including proinflammatory changes and an increased expression of cell adhesion molecules, whereas preclinical data on vascular and hemostatic effects of gemcitabine remain scarce and inconclusive [12,13,14,15].

Nonetheless, our findings suggest that the high burden of VTE in patients treated with platinum agents or gemcitabine are possibly not be fully causal, but to a large degree be mediated by underlying prothrombotic risk factors that are correlated with treatment with these agents. For example, gemcitabine is one mainstay of treatment for pancreatic cancer, and platinum-based agents are a treatment standard for gastric cancer, and both these tumor entities are associated with the highest known risk of VTE [16,17].

A limitation of our analysis is that treatment with platinum-based agents and gemcitabine was not analyzed as a time-dependent variable and applied cumulative doses were not considered. Although time-dependent and dose-specific analyses could have yielded a more refined appreciation of the association between these treatments and cancer-associated VTE risk, time-dependent variables are not useful for clinical prediction purposes as their information only becomes available over time, and thus cannot be used for clinical decision making.

## 4. Methods

This analysis was conducted within a prospective, observational cohort study, the Vienna Cancer and Thrombosis Study (CATS). The design, procedures, as well as inclusion and exclusion criteria have been previously reported in detail [18], and the study was approved by the Ethics Committee of the Medical University of Vienna (Project identification code: 126/2003, date of approval: 2 September 2003). Briefly, patients with newly diagnosed cancer or recurrent or progressive disease after remission were eligible for inclusion. Patients were followed prospectively for the occurrence of objectively confirmed, independently adjudicated VTE for a maximum of 2 years.

We used the same dataset as the CATS model derivation study (*n* = 1423) [8], with risk of VTE stratified according to tumor type, i.e., very high risk (pancreas and gastric), high risk (lung, colorectal, oesophagus, kidney, lymphoma, bladder/urothelial, uterus, cervical, ovarian, and others), and intermediate/low risk (breast and prostate). D-dimer levels were obtained from blood withdrawn at study inclusion and measured with the STA-Liatest assay (Diagnostica-Stago, Asnières, France). Data on chemotherapy during the 2-year observation period were obtained from the in-house pharmacy prescription records, with missing chemotherapy data in 14 patients (analysis cohort, *n* = 1409).

### Statistical Analysis

Baseline differences between groups were analyzed by means of the rank sum test or Chi-square test, as appropriate. Between-group differences in risk of VTE were assessed with competing risk cumulative incidence estimators and Fine and Gray proportional subdistribution hazards regression models [19,20]. A sensitivity analysis was conducted with the most recent dataset available from CATS, consisting of 1870 patients.

## 5. Conclusions

Therapy with gemcitabine was not associated with an increased risk of VTE. Platinum-based therapy could be a weak independent risk factor for cancer-associated VTE, with only limited predictive value beyond tumor site category and D-dimer levels. These findings do not support the use of these variables in clinical prediction models for VTE in patients with cancer.

## Figures and Tables

**Table 1:** Baseline characteristics of study cohort.

Variable	Overall (*n* = 1409)	No Gemcitabine (*n* = 1174)	Gemcitabine (*n* = 235)	*p* ^1^	No Platinum (*n* = 908)	Platinum (*n* = 501)	*p* ^1^
Clinical Variables							
Age at entry (years)	62.9 (54.2–68·9)	63.0 (53.6–68·9)	62.6 (56.1–69.6)	0.256	63.7 (53.9–70.4)	61.2 (54·4–67.2)	0.003
BMI (kg/m^2^)	25.1 (22.1–28.3)	25.3 (22.5–28.5)	24.0 (20.9–26.5)	<0.0001	25.4 (22.5–28.7)	24.5 (21.6–27.3)	<0.0001
Male sex	760 (54%)	640 (55%)	120 (51%)	0.333	472 (52%)	288 (57%)	0.047
Tumor site							
Low/intermediate risk of VTE	378 (27%)	366 (31%)	12 (5%)	<0.0001	373 (41%)	5 (1%)	<0.0001
Breast	226 (16%)	215 (18%)	11 (5%)	<0.0001	224 (25%)	2 (0%)	<0.0001
Prostate	153 (11%)	151 (13%)	1 (<1%)	<0.0001	149 (16%)	3 (1%)	<0.0001
High Risk of VTE	854 (61%)	733 (62%)	121 (51%)	<0.0001	467 (51%)	387 (77%)	<0.0001
Lung	289 (21%)	213 (18%)	76 (32%)	<0.0001	88 (10%)	201 (40%)	<0.0001
Colorectal	171 (12%)	170 (14%)	1 (<1%)	<0.0001	68 (7%)	103 (21%)	<0.0001
Kidney	42 (3%)	33 (3%)	9 (4%)	0.401	39 (4%)	3 (1%)	<0.0001
Lymphoma	247 (18%)	241 (21%)	6 (3%)	<0.0001	227 (25%)	20 (4%)	<0.0001
Other sites	105 (7%)	76 (6%)	29 (12%)	0.002	45 (5%)	60 (12%)	<0.0001
Very high risk of VTE	177 (13%)	75 (6%)	102 (43%)	<0.0001	68 (7%)	109 (22%)	<0.0001
Pancreas	116 (8%)	14 (1%)	102 (43%)	<0.0001	49 (5%)	67 (13%)	<0.0001
Stomach	61 (4%)	61 (5%)	0 (0%)	<0.0001	19 (2%)	42 (8%)	<0.0001
Tumor characteristics							
Newly diagnosed cancer	997 (71%)	819 (70%)	178 (76%)	0.066	606 (67%)	391 (78%)	<0.0001
Tumor grade G3/G4	518 (38%)	418 (36%)	100 (43%)	0.05	315 (36%)	203 (41%)	0.04
Tumor stage (UICC/AnnArbor)	/	/	/	<0.0001	/	/	<0.0001
Stage I	138 (10%)	133 (12%)	5 (2%)	/	116 (14%)	22 (4%)	/
Stage II	309 (23%)	278 (25%)	31 (13%)	/	264 (31%)	45 (9%)	/
Stage III	221 (16%)	184 (17%)	37 (16%)	/	124 (15%)	97 (19%)	/
Stage IV	672 (50%)	511 (46%)	161 (69%)	/	336 (40%)	336 (67%)	/
Biomarker levels							
D-dimer (µg/mL)	0.7 (0.4–1.5)	0.7 (0.3–1.3)	1.2 (0.6–2.6)	<0.0001	0.6 (0.3–1.2)	1.0 (0.5–2.0)	<0.0001
VTE prediction model							
CATS score: predicted 6-month VTE risk (%) ^2^	5.0 (3.3–6.3]	4.8 (2.8–5.70	7.4 (5.2–11.0)	<0.0001	4.6 (2.6–5.5)	5.8 (5.0–8.6)	<0.0001
Outcomes							
Mortality	532 (37.8%)	377 (32.1%)	155 (66.0%)	/	253 (27.9%)	279 (55.7%)	/
VTE events	111 (7.9%)	87 (7.4%)	24 (10.2%)	/	53 (5.9%)	58 (11.6%)	/

Continuous variables are summarized by median and corresponding interquartile range and categorical data by absolute frequency and percentages. ^1^ *p*-Value from rank-sum test or Chi-square test (as appropriate) for the comparison of baseline variables between gemcitabine vs. no gemcitabine and platinum vs. no platinum therapy. ^2^ Based on tumor site risk category and continuous levels of D-dimer. BMI, body mass index; UICC, Union for International Cancer Control; VTE, venous thromboembolism.

**Table 2:** Baseline characteristics of study cohort.

Model	Variable	SHR	95%CI	*p*
**#1**	Gemcitabine	0.83	0.53–1.31	0.432
	Tumor type: Low/moderate VTE risk	Ref.	Ref.	Ref.
	Tumor type: High VTE risk	2.48	1.37–4.48	0.003
	Tumor type: Very high VTE risk	5.48	2.82–10.68	<0.0001
**#2**	Gemcitabine	1.18	0.75–1.85	0.481
	D-dimer (per doubling)	1.44	1.25–1.66	<0.0001
**#3**	Gemcitabine	0.82	0.53–1.28	0.390
	Tumor type: Low/moderate VTE risk	Ref.	Ref.	Ref.
	Tumor type: High VTE risk	2.26	1.24–4.10	0.008
	Tumor type: Very high VTE risk	4.27	2.10–8.66	<0.0001
	D-dimer (per doubling)	1.31	1.11–1.53	0.001
**#4**	Platinum-based therapy	1.46	0.97–2.21	0.073
	Tumor type: Low/moderate VTE risk	Ref.	Ref.	Ref.
	Tumor type: High VTE risk	2.03	1.09–3.77	0.025
	Tumor type: Very high VTE risk	3.89	1.87–8.11	<0.001
**#5**	Platinum-based therapy	1.84	1.26–2.69	0.002
	D-dimer (per doubling)	1.40	1.20–1.63	<0.0001
**#6**	Platinum-based therapy	1.44	0.96–2.17	0.080
	Tumor type: Low/moderate VTE risk	Ref.	Ref.	Ref.
	Tumor type: High VTE risk	1.85	0.99–3.46	0.055
	Tumor type: Very high VTE risk	3.08	1.45–6.57	0.004
	D-dimer (per doubling)	1.31	1.1–1.53	0.001

Multivariable competing risk models of gemcitabine therapy, platinum-based therapy, tumor type, and D-dimer for prediction of VTE in patients with cancer (*n* = 1409). 95% CI, 95% confidence interval; *p*, Wald test *p*-value; Ref., reference category; SHR, subdistribution hazard ratio; VTE, venous thromboembolism.
